# A Volunteer Surgeon in War Zones: Experience of 35 Years and a Call to Action

**DOI:** 10.1007/s40719-017-0075-1

**Published:** 2017-02-08

**Authors:** Christos Giannou

**Affiliations:** 1 Queen Mary and Barts School of Medicine and Dentistry, Blizard Institute, University of London, London, UK; 2 National Critical Care and Trauma Response Centre, Royal Darwin Hospital, Darwin, Australia

… he who desires to practice surgery must go to war

Hippocrates (460 – 377 BCE)

The aphorism of Hippocrates has become a cliché. From Larrey to Pirogov, Winnett-Orr and Trueta to DeBakey, the cliché has been proven to be a reality. The idea that surgery during armed conflict advances our knowledge of medicine and surgery has become commonplace. After 35 years’ experience, I think back on some of the implications of these clichés and commonplace ideas.

Some progress has been due to technological developments, some to a better understanding of pathophysiology, and some simply due to the development of more efficient techniques. More than anything, the improved organisation of medical services should not be underestimated. This is especially the case in prehospital first aid. Indeed, it has been shown that good first aid and resuscitation decreases mortality more than what might be called “precocious” emergency surgery [1]. Nonetheless, and despite the technical progress, I think that many “old-fashioned” techniques are still best.

There is not, of course, only one school of surgery practiced in time of armed conflict. Four different distinct levels of sophistication can be defined and described depending on the socioeconomic development of the country in question as well as other external constraints of the environment [2]. Surgical techniques and organisation of medical services must be adapted to each specific situation.

1. For the conventional army of an industrialised society, often nothing less than a level I trauma centre has become the accepted norm. This level of service includes the rapid evacuation and transfer of patients.
2. In an emergent developing society, there may be a high level of specialised surgical care, at least in the major cities if not in remote rural areas. Projection of this surgical capacity to the field is irregular. Evacuation of patients is possible, if sometimes difficult.
3. In a poor country with limited financial and human resources, a few major surgical facilities may exist in the capital city, but little exists elsewhere. In general, supplies, equipment, budgets and personnel are chronically inadequate or even absent. Evacuation of patients is difficult or impossible.
4. In extreme situations, safe access to the victims by health professionals, and the victims’ access to medical care, is impossible or rare and always a challenge. This situation involves populations without safe access to public structures (one thinks of present-day Syria or Yemen): non-state actors and guerrilla groups. Field surgery, when practised, is carried out by a few trained doctors and nurses under precarious conditions because there is no alternative.

Most war-wounded in contemporary conflicts are not managed by a well-financed classical army with an abundance of personnel, equipment and supplies. Most are treated in local civilian hospitals in developing and poor countries by medical teams that often have difficulty in coping with the needs of their patients in the best of times. And even if, at the best of times, hospital services can offer adequate surgical treatment, the circumstances of conflict quickly degrade those capacities: damaged or destroyed infrastructure; problems with equipment (maintenance, repair, spare parts); lack of renewable supplies and medicines; dysfunctional administration; and personnel who may be killed or injured, or paralysed by fear, or become refugees, or may work for months on end with no payment of salaries. Surgery for the victims of armed conflict as practiced by most humanitarian agencies tries to address the challenges of the last two categories cited above, under conditions whereby the very first victim of war is the health system.

… give succour, and do no harm

Hippocrates

Humanitarian surgery is a surgery of challenges, above and beyond that of a precarious or even dangerous environment. Special rules apply: international humanitarian law (IHL): the Geneva Conventions of 1949 for the protection of the sick and wounded and of those who care for them. But how to make combatants comply with IHL? There is a specific epidemiology of war wounds, with a predominance of emergency surgery, but undertaken in a limited technical environment, often with scarce resources. Even an experienced trauma surgeon will be challenged by the wound pathology caused by weapons—bullets, bombs, blast, and non-conventional weapons—with specific techniques that are appropriate to the context and pathology. This is septic surgery, consisting of debridement and delayed primary closure with no internal fixation of fractures. These circumstances are not new to an older generation of surgeons and those trained and who work in the Third World, but they are unfamiliar to newer generations. These are simple, safe, and rapid techniques.

Unlike natural disasters, where all wounded patients are brought about at one point in time, mass casualties involving the principles of triage during armed conflict are faced day after day after day, until the cessation of hostilities. This involves a special personnel roster and re-organisation of the functioning of the hospital. With true mass casualties, there is a category of patient labelled “leave to die in peace and with dignity,” probably the most difficult decision in medicine. There is also a category of absolute urgency when a young combatant under the influence of a “toxic cocktail”—a mixture of adrenaline, testosterone, alcohol, cannabis, amphetamines etc.—points a rifle between your ribs and announces: “take care of my friend first”. This is a situation not mentioned in standard textbooks.

What this means practically in a context of limited resources—humanitarian and civilian surgery, categories three and four above—is that whole fresh blood rather than blood components is the norm; a strict transfusion protocol of which patients are to receive blood, rather than a massive transfusion protocol, is standard practice; baseline anaesthesia is by ketamine injection; and damage control surgery is rare, because one may never have the opportunity of seeing the same patient again. Diagnosis depends on the “eye, ear, nose and ten-finger whole-body scan”: good and complete clinical examination is the foundation of diagnosis; consider yourself lucky if the X-ray machine is working. It is the lack of technology—diagnostic and therapeutic—even more than the pathology of weapon wounds that is the greatest challenge to the newer generation of surgeons. Indeed, the attending physician must all too often be a very general, general surgeon, covering all surgical subspecialties, including emergency obstetrics: there is usually no gynaecologist around to perform a Caesarean section, yet another challenge to be faced in an age of precocious hyper-specialisation.

Wherever the art of medicine is loved, there also is love for humanity.

Hippocrates

Beginning in the 1970s, an idealistic generation that came out of the worldwide contestation of the established order entered humanitarian work *en masse*. The best known group of this phenomenon was Médecins sans Frontières (MSF: Doctors Without Borders), who joined their colleagues of the International Committee of the Red Cross (ICRC) in the field. Soon, many other non-governmental organisations were created. There was great enthusiasm, but most programmes involved the “substitution” of local structures, often deficient or simply absent, by foreign humanitarian organisations.

The world has moved on since. The Cold War ended, conflicting ideological tensions decreased, but old demons came back to life. Identity politics and their ideological manifestation—nationalism, clannism, religious intolerance—came to the fore again. Newer generations of medical professionals arrived whose livelihoods were no longer as guaranteed as before, and governments that had encouraged humanitarian work stopped being so accommodating and generous. Educational progress in many countries meant that there was no longer a dearth of doctors and nurses. In 2000, the city of Kisangani in Democratic Republic of the Congo witnessed a series of battles. I arrived as an ICRC surgeon to find three functioning hospitals with six surgeons who had been trained in Paris or Brussels working away. These doctors had completed their training and rather than remaining in Europe, they returned home to serve their own people. The pathology was new for them; my work consisted in a war surgery training seminar and then operating together to acquaint them with the techniques appropriate to wounds caused by the weapons of war. Substitution gave way to “support”.

In spite of impressions due to a 24-h news cycle, there are actually fewer armed conflicts today than a generation ago. The needs continue nonetheless. Access has become more difficult; the security of humanitarian workers in general, and medical teams in particular, more problematic. Recent examples include the assassination of six ICRC medical staff in a hospital in Chechnya in December 1996–20 years already!—and the bombing of MSF hospitals in Afghanistan, Yemen and Syria, and public hospitals in general in those same countries along with South Sudan. The challenge of combatant compliance with IHL remains. War is never the “good guys” versus the “bad guys”, but rather the bad guys against the worse; even good people do evil in wartime. How easily the restraints of what we call civilisation can fall apart.

The practice of medicine is already a vocation, a calling, and not a simple profession, unless it be a profession of faith and commitment. Surgery is demanding; emergency and trauma surgery demand sacrifices of one’s private life, of one’s empathy. A trauma surgeon must be able to go from death bed—a failure to extend the life of an otherwise healthy trauma victim—to operating table. Surgery during armed conflict compounds the challenges, the sacrifices, the psychological dissociation necessary to cut into human flesh, all the while controlling one’s fear from ongoing nearby combat. Yet, working alongside national colleagues who remain and who put themselves and their families in danger to take care of the victims of armed conflict is humbling and exhilarating. Yes, the challenges are great; the exhilaration and gratification of helping those most in need cannot be described on the written page. That is why we go back again and again.
